# Surface Engineering for PMMA-Based Removable Prostheses: A Narrative Review

**DOI:** 10.3390/polym18141765

**Published:** 2026-07-20

**Authors:** Jamal Al Ashkar, Nicoleta Ioanid, Delia Teodora Dima, Ruxandra Teodora Stan, Andreas Katsonis, Ana-Maria Raluca Pauna, Roxana-Ionela Vasluianu

**Affiliations:** Grigore T. Popa University of Medicine and Pharmacy, 700115 Iasi, Romania; jamal.al-ashkar@d.umfiasi.ro (J.A.A.); nicoleta.ioanid@umfiasi.ro (N.I.); delia-teodora.dima@d.umfiasi.ro (D.T.D.); ruxandra-teodora.stan@umfiasi.ro (R.T.S.); paunaanamariaraluca@gmail.com (A.-M.R.P.); roxana.vasluianu@umfiasi.ro (R.-I.V.)

**Keywords:** polymethylmethacrylate, hydroxyapatite, silica, titanium dioxide, removable prosthodontics, ceramics, antibacterial, osteoconduction

## Abstract

Polymethylmethacrylate (PMMA) is still the most widely used prosthetic polymer, although its biological inertness and vulnerability to mechanical stress and microbiological colonization are gradually restricting its therapeutic lifespan. This narrative review develops a conceptual framework, three ceramic modifiers corresponding to three distinct biofunctional strategies, to logically guide the design of the next generation of PMMA-based prosthetic dentures. We critically analyze the transformation of hydroxyapatite (HA), silica (SiO_2_), and titanium dioxide (TiO_2_) from passive fillers to active functional phases, offering unique, complementary therapeutic advantages. Therefore, HA confers osteoconductive and bone affinity, SiO_2_ provides surface reactivity, tunable bioactivity, and drug release capacity, while TiO_2_ provides mechanical reinforcement, chemical stability, and photocatalytic antibacterial activity. These ceramics used in PMMA matrices result in hybrid materials that outperform standard resins in terms of structural, mechanical, and biological performance. Recent research on binary and ternary systems (e.g., HA–TiO_2_, SiO_2_–HA, and HA–SiO_2_–TiO_2_ in PMMA) has indicated synergistic effects, such as increased osteoblast proliferation, reduced biofilm development, improved fracture toughness, and favorable corrosion resistance in simulated oral environments. A decision matrix is also provided to assist the clinician in selecting the best ceramic for a given clinical function of a prosthetic base or provisional repair. Although polymer–ceramic hybrid systems show remarkable translational potential, there are still obstacles to be addressed in terms of long-term interfacial stability, standardized synthesis processes, and regulatory mechanisms. This review proposes a framework of PMMA as a multimodal biofunctional engineering platform rather than a basic structural polymer and provides a roadmap for the development of intelligent, interactive, and clinically durable prosthetic materials.

## 1. Introduction

For decades, polymethylmethacrylate (PMMA) has been the base of prosthetic practice, being appreciated for its low cost, good esthetics, ease of processing and clinical familiarity [1,2,3]. Advances in biomaterials science and surgical procedures, together with increasing patient expectations, have transformed once acceptable compromises into unacceptable clinical deficiencies.

A principal limitation of unmodified PMMA is not mechanical but biological. When in contact with the oral mucosa, gingiva, or alveolar bone, PMMA remains chemically and physically inert. This inertness prevents acute toxicity or inflammation, but it also fails to bring significant improvements to the host. Histological studies consistently demonstrate that PMMA prostheses are surrounded by a non-adherent collagenous fibrous capsule, rather than integrating with the underlying tissue. This is not true healing, but a foreign body response.

In patients wearing the prosthesis, the fibrous layer acts as a lubricated interface, allowing movement of the prosthesis relative to the mucosa, thus compromising fit and comfort. Without biological adhesion, functional loads are transmitted through discrete pressure points rather than being distributed across a bonded interface, and diminished sensory feedback primarily affects masticatory efficiency. An important aspect is that in implant-prosthetic therapy, PMMA’s inability to adhere to bone prevents its use as an osseointegrated, load-bearing material, being placed in temporary restorations or denture bases that are overlying healed ridges [1,3].

Microcracks accumulate under repeated masticatory loads, leading to unpredictable failures after months of apparently satisfactory use [3,4,5]. Water absorption (1.5–2.5% by weight) acts as a contributing factor to degradation, causing dimensional changes, plasticization that decreases modulus and strength, monomer leaching, and interfacial delamination in repaired dentures [6,7,8]. The cumulative effect is a denture that becomes progressively weaker and less stable over time, not only due to aging but also due to intrinsic degradation caused by the oral environment [5,7].

Perhaps the most burdensome clinical consequence is the clinically significant susceptibility of PMMA to biofilms. Candida albicans readily adheres to the hydrophobic and irregular surface of PMMA, maturing into a three-dimensional biofilm. The result is palatal inflammation and erythema, affecting 40% to 70% of complete denture wearers [4,9,10]. In response to these deficits, a new generation of hybrid materials is emerging, designed not only to replace lost tissue but also to actively participate in the biological and mechanical environment of the oral cavity [2,3].

This narrative review proposes a conceptual framework in which hydroxyapatite (HA), silica (SiO_2_), and titanium dioxide (TiO_2_) are integrated into PMMA not as passive fillers but as functional bioactive phases. Such hybrids aim to improve not only mechanical longevity but also biological integration, antimicrobial performance, and overall tissue response, critical requirements in an environment characterized by cyclic loading, humidity, thermal fluctuations, and microbial challenge. The present article critically reviews the synthesis, properties, and translational potential of PMMA systems modified with HA, SiO_2_, and TiO_2_, with a focus on their role in advanced prosthetics, and concludes with a discussion of current challenges and future directions toward biofunctional and clinically transferable PMMA-based prostheses. We specifically exclude direct restorative materials and fixed provisional crowns, where different performance requirements apply.

## 2. Materials and Methods

### 2.1. Search Strategy

A systematic literature search was performed in PubMed, Web of Science and Scopus for publications from January 2000 to April 2026. The search combined keywords for PMMA (“polymethylmethacrylate”, “acrylic resin”, “denture base”), ceramic modifiers (“hydroxyapatite”, “HA”, “silica”, “SiO_2_”, “titanium dioxide”, “TiO_2_”, “nanoparticle”) and application (“denture”, “removable”, “interim”, “temporary”). The reference lists of included articles and relevant systematic reviews were hand-searched for additional studies. The initial search identified 1247 records in PubMed, 982 in Web of Science, and 1104 in Scopus. After removing duplicates, 2891 unique records remained for screening. Although this is a narrative rather than a systematic review, we adopted a PRISMA-based approach to improve transparency and reproducibility.

### 2.2. Inclusion and Exclusion Criteria

Studies that evaluated PMMA modified with HA, SiO_2_ or TiO_2_ for removable or intermediate dentures, reported mechanical, surface, antimicrobial or biocompatibility results, were original in vitro, in vivo or clinical research, and studies that were published in English were included. Exclusion criteria were: fixed prostheses, non-ceramic modifiers (e.g., silver, zinc oxide alone), editorials, conference abstracts and publications in non-English.

### 2.3. Study Selection and Data Extraction

Titles and abstracts were selected, followed by a full-text review. Data extraction was performed based on material composition, particle characteristics, manufacturing method, characterization techniques, aging protocols, and quantitative results.

## 3. Three Ceramics with Distinct Biofunctional Roles

The strategic addition of ceramic phases to PMMA represents a shift from passive reinforcement to active biofunctionalization. Three ceramics have been identified as the most promising modifiers for removable prostheses: HA, SiO_2_, and TiO_2_. Each ceramic has unique physicochemical properties, which result in complementary biofunctional mechanisms (Table 1).

### 3.1. Hydroxyapatite (HA): Biomimetic Osteoconduction and Tissue Affinity

#### 3.1.1. Fundamental Properties and Rationale

Hydroxyapatite [Ca_10_(PO_4_) _6_(OH)_2_] is the most thermodynamically stable phase of calcium phosphate under physiological conditions, showing crystallographic and chemical similarity to the mineral component of human bone and enamel. This biomimetic property underlies its exceptional biocompatibility and osteoconductivity. When placed in contact with living bone, HA supports the adhesion, proliferation and differentiation of osteoblasts, promoting de novo bone formation directly on its surface, without an intermediate fibrous layer [2,3].

For removable prosthetic applications, the value of HA is predominantly concentrated on the surface. Instead of loading the material, which risks stress concentration, HA is optimally applied as thin enriched layers, surface coatings or modifications within PMMA with low loading (typically 1–5 wt%). This structure is intended to improve mucosal compatibility, reduce localized wear, or act as a carrier for antimicrobial ions [11,12,13].

#### 3.1.2. Mechanistic Basis of Bioactivity

**Figure 1:**
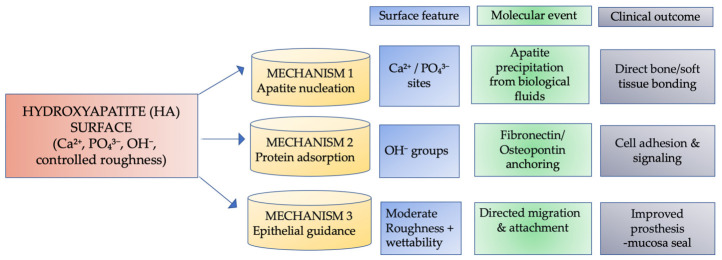
The bioactivity of hydroxyapatite.

The bioactivity of HA arises from three interconnected mechanisms, represented by:

1. Surface calcium and phosphate ions facilitate the nucleation of apatite from surrounding biological fluids, creating a mineralized interface that can bind to bone or soft tissue [29].
2. Surface hydroxyl groups (-OH) provide sites for the adsorption of proteins, particularly fibronectin and osteopontin, which mediate cell adhesion and signaling [2].
3. The moderate surface roughness and favorable surface energy of HA promote migration and attachment of epithelial cells, potentially improving the seal at the prosthesis–mucosal interface (Figure 1).

#### 3.1.3. Critical Determinants of Performance

The performance of HA-modified PMMA is highly sensitive to particle size, dispersion quality, and interfacial chemistry. Pan et al. demonstrated that PMMA-grafted HA significantly increased surface hardness and elastic modulus compared to untreated HA, which was attributed to better stress transfer through a continuous interphase [11]. Aldabib and Ishak systematically adjusted the HA content (0–15 wt%) and showed that low concentrations (≤5 wt%) of HA improved mechanical properties, with higher loadings resulting in agglomeration and stress concentration, leading to lower transverse strength and fatigue life [13]. Fouly et al. extended these observations to tribological behavior, reporting that low HA loading fractions (1–3 wt%) increased wear resistance and surface hardness without unacceptably increasing water sorption [12]. However, they cautioned that particle aggregation, especially micro-sized HA, caused three-body abrasive wear mechanisms that exacerbated surface damage. Shahkar et al. investigated hybrid reinforcement using HA with multi-walled carbon nanotubes (MWCNTs) in PMMA denture bases [30]. The HA-MWCNT combination provided synergistic mechanical reinforcement (increases in flexural strength of ~40% and elastic modulus of ~55%), although the authors stated that the primary utility of HA was as a biofunctional platform rather than as the primary mechanical reinforcement. The nanotubes acted as a mechanical framework, while HA provided bioactivity, a design idea that has broader implications for hybrid systems.

#### 3.1.4. Ion-Doped HA: Designing Antimicrobial Function

Pure HA possesses negligible direct antimicrobial activity. However, its crystal lattice readily accepts ionic substitutions, allowing the incorporation of antimicrobial metal ions. Saskianti et al. synthesized HA-PMMA nanocomposites doped with silver (Ag) and titanium (Ti) and evaluated their activity against Streptococcus mutans, Candida albicans, and Staphylococcus aureus. The doped systems demonstrated significant inhibition zone diameters (12–18 mm) compared to pure HA controls (0 mm), with Ag-doped HA exhibiting the strongest activity. Critically, the antimicrobial effect was surface-dependent. Studies confirmed sustained ion release over 14 days, but the authors noted that controlled release engineering, including encapsulation strategies or mesoporous delivery systems, would be required for sustainable clinical performance beyond short-term provisional use [14].

#### 3.1.5. Clinical Translation Recommendations for HA-PMMA

Available in vitro data suggest that surface application, rather than bulk incorporation, is the most effective way to achieve the biofunctional benefits of HA. Surface coatings or thin films enriched with HA (typically 1–5 μm thick) can provide bioactivity at the tissue interface while preserving the flexural strength of the PMMA matrix [11,13]. However, significant reductions in flexural strength (20–35% in some studies), stress concentration, and particle agglomeration have been associated with bulk incorporation of HA particles, especially at levels greater than 5 wt% [12,13].

For in vitro mechanical investigations, HA nanoparticles with a size range of 20–100 nm have an ideal HA loading range of 1–5 wt%. When these particles are uniformly distributed, they can improve surface hardness and elastic modulus without sacrificing flexural strength [13,15]. Further evidence suggests:

Nano-HA (20–100 nm) is preferred over micro-HA due to the smaller particles that reduce stress concentration effects and provide a larger surface area for biofunctional interactions [13].

- Surface modification (e.g., silanization, PMMA grafting) appears to be a requirement for uniform dispersion and sufficient interfacial adhesion between HA particles and the hydrophobic PMMA matrix [11,15].
- Higher loadings (>5–7 wt%) consistently lead to particle agglomeration, porosity, and diminished mechanical performance in vitro [12,13].

As a clinical potential, we can highlight the following aspects:

- The main biofunctional mechanism of HA (osteoconduction through apatite nucleation) has limited relevance for conventional denture bases covering keratinized oral mucosa [3].
- Pure HA possesses negligible direct antimicrobial activity. Antimicrobial functionality requires ionic doping (Ag^+^, Ce^4+^, Ti^4+^) with the additional variables of controlled release and potential cytotoxicity [14].
- The clinical significance of HA-induced improvements in mucosal compatibility or tissue response is unclear.

### 3.2. SiO_2_: Surface Reactivity and Mesoporous Drug Delivery

#### 3.2.1. Dual Functionality: Mechanical Modifier and Antimicrobial Carrier

SiO_2_ offers two complementary benefits for PMMA modification: mechanical tuning and mesoporous antimicrobial drug delivery. Unlike HA, which primarily confers bioactivity, SiO_2_ can simultaneously enhance mechanical properties and serve as a refillable drug reservoir, a combination that has attracted substantial research interest [16,17,18].

#### 3.2.2. Mechanical Strengthening by Silanization

The key to the mechanical contribution of SiO_2_ lies in its interfacial chemistry. Unmodified SiO_2_ particles have poor compatibility with the hydrophobic PMMA matrix, leading to agglomeration, porosity and reduced mechanical properties. Silanization, the covalent attachment of organosilane coupling agents (e.g., 3-(trimethoxysilyl)propyl methacrylate), creates a chemical bridge between the SiO_2_ surface (via Si-OH groups) and the methacrylate polymer matrix (via C=C bonds), as schematically shown in Table 2.

A thorough evaluation of silanized nanosilica in PMMA for fixed provisional prostheses by Topouzi et al. showed a considerable improvement in flexural strength (up to 38%), surface hardness (up to 27%), and reduced porosity compared to unsilanized controls [31]. Jiangkongkho et al. demonstrated that controlled dispersion of silanized nanosilica (1–3 wt%) produced adequate mechanical properties without affecting the manageability or esthetics of prosthetic restorations [20]. Higher loadings (≥5 wt%) resulted in agglomeration, higher viscosity, and lower flexural strength.

Alzayyat et al. also performed similar in vitro studies on SiO_2_-reinforced denture base resins and confirmed that 3 wt% silanized nanosilica improved flexural strength by approximately 25% and decreased surface roughness (Ra values from 0.32 µm to 0.18 µm) [32,33]. Al-Thobity et al. performed a comprehensive review and meta-analysis and found that appropriately silanized SiO_2_ nanoparticles consistently increased the flexural strength of thermally cured PMMA, with a cumulative effect size of approximately 22% [21].

#### 3.2.3. Mesoporous Silica: Reloaded Antimicrobial Nanocarriers

The most important application of SiO_2_ in PMMA prostheses is in the form of mesoporous nanocarriers, highly porous SiO_2_ nanoparticles (pore width 2–50 nm) capable of loading and releasing therapeutic chemicals [16,17,20]. Mesoporous silica nanoparticles (MSNs) have several benefits for antimicrobial delivery, namely:

- High loading capacity—Pore volumes of 0.5–1.5 cm3/g allow loading of 20–40% by weight of antibacterial agents [16].
- Controlled release—Release kinetics from hours to weeks are controlled by the pore architecture (size, connectivity, surface functionalization) [20].
- Rechargeable—MSNs can be re-exposed to antimicrobial solutions for recharge after depletion, leading to maintenance of clinical function [17].
- Matrix preservation—The antibacterial action is localized to the surface of the nanoparticles and in the pores, so the overall mechanical capabilities of the PMMA matrix are not affected [16].

Lee et al. fabricated PMMA with mesoporous SiO_2_ nanocarriers loaded with chlorhexidine and demonstrated sustained release of the antimicrobial for 30 days without degradation [16]. The scientists also demonstrated the efficacy of the device following thermal cycling (5000 cycles, 5–55 °C), which simulated intraoral aging.

Jo et al. proposed this approach and developed a rechargeable microbial antiadhesive PMMA with silver sulfadiazine-loaded MSNs [17]. Once the initial antimicrobial load was depleted, the dentures were placed in a silver sulfadiazine solution and successfully recharged for three additional 14–21-day cycles of antibacterial activity. This rechargeable paradigm is a far cry from traditional antibacterial compounds that tend to become less effective over time. Hata et al. found that the pore design, especially pore volume and connectivity, of nanoporous SiO_2_ fillers in PMMA controlled the kinetics of water sorption and release [20]. Optimal pore widths (10–20 nm) were reported as a compromise between high loading capacity and controlled release, while larger pores (>50 nm) resulted in burst release and increased water sorption.

### 3.3. TiO_2_: Mechanical Reinforcement and Photocatalytic Antimicrobial Activity

#### 3.3.1. The Most Versatile Ceramic Modifier

TiO_2_ has emerged as the most potent ceramic modifier for PMMA in removable prostheses, providing mechanical reinforcement and surface antibacterial activity [23,24,27,28].

TiO_2_ is unique in combining with HA (mostly osteoconductive) or SiO_2_ (mostly mechanical/transmission) and offers the following benefits:

- Reinforcement phase with high inherent strength and elastic modulus [22,34];
- Photocatalytic production of reactive oxygen species (ROS) under ultraviolet (UV) or visible light [35,36];
- Chemical stability in oral fluids with little or no leaching or degradation [23];
- Adequate surface energy to promote protein adsorption and cell adhesion in some cases [24].

#### 3.3.2. Mechanical Strengthening: Anatase Phase and Particle Morphology

In 2025, in a comprehensive evaluation, Melo-Soares et al. showed that nanoscale TiO_2_ in the anatase phase produced the best consistent antibacterial and mechanical results [27]. The rutile phase, however, is more thermodynamically stable and has reduced photocatalytic activity and mechanical consistency of the reinforcement. Totu et al. demonstrated the feasibility of incorporating TiO_2_ nanoparticles into a PMMA-based resin for stereolithography (SLA) 3D printing of complete dentures, while Chen et al. reported that the addition of TiO_2_ (2–5 wt%) significantly improved the mechanical properties of 3D printed PMMA composite resin, including enhanced flexural strength and impact resistance [22,37]. Liao et al. extended this to digital light processing (DLP) printing, demonstrating that reinforcement with TiO_2_ and polyetheretherketone (PEEK) resulted in a 3D printable PMMA composite with mechanical properties comparable to those of conventionally processed materials [38].

Particle shape is very important for mechanical performance. Abdulrazzaq Naji et al. compared TiO_2_ nanoparticles (20–40 nm) and TiO_2_ nanotubes (length 80–120 nm, diameter 10–15 nm) hydrothermally synthesized and implanted into PMMA [25,26]. Nanotubes offered higher fracture toughness (52% vs. 28% for nanoparticles) and microhardness (35% vs. 18%) due to mechanical interlocking and better stress transmission through the polymer–nanotube interface.

Kumar et al. and Pai et al. also studied individual TiO_2_ and silver nanoparticles (AgNPs) in PMMA and demonstrated that TiO_2_ showed comparable or better improvements in flexural strength (20–25% versus 10–15% for AgNPs) and improved color stability [39,40]. This makes TiO_2_ the preferred material for combining mechanical strengthening with antibacterial activity.

#### 3.3.3. Mechanism of Antimicrobial Photocatalysis

TiO_2_ has antimicrobial properties due to its photocatalytic activity. Upon irradiation with light with an energy higher than the band gap (~3.2 eV for anatase, i.e., UV light at λ < 387 nm), electron–hole pairs are generated in TiO_2_ that interact with adsorbed water and oxygen to generate reactive oxygen species (ROS), such as hydroxyl radicals (•OH), superoxide anions (O_2_•−), and hydrogen peroxide (H_2_O_2_) [27,28].

Giti et al. studied PMMA reinforced with 2–3 wt% TiO_2_ nanoparticles and found a significant reduction in the viability of C. albicans and S. aureus under UV irradiation (365 nm, 2 h) [35]. An increase in antifungal activity was observed with increasing TiO_2_ levels up to 5 wt%. The authors also found that the antibacterial effect was very weak in the dark. Therefore, TiO_2_ photocatalysis is sensitive in certain situations [35]. In 2024, Altarazi et al. also used this method for nanocomposite resins for 3D printed denture bases, where they showed that the addition of TiO_2_ (3 wt%) reduced C. albicans biofilm generation by more than 70% under simulated oral lighting circumstances (ambient light, 12 h cycles) [36]. Thermocycling (10,000 cycles, 5–55 °C) did not influence the antibacterial activity, demonstrating an acceptable retention of nanoparticles in the polymer matrix.

#### 3.3.4. Composites Using TiO_2_

Hybrid systems combining TiO_2_ with other phases have been investigated for synergistic effects. Liu et al. reported that PMMA modified with TiO_2_-HAP demonstrated photodynamic antibacterial activity against C. albicans (99% reduction in 30 min) without cytotoxicity [41]. Cascione et al. blended TiO_2_ with halloysite clay nanotubes, achieving improved mechanical properties and antibacterial activity through complementary strengthening mechanisms [42].

Based on in vitro evidence, several preliminary design considerations can inform the development of TiO_2_-PMMA composites. The anatase phase, in the form of nanoparticles (20–50 nm) or nanotubes (10–15 nm diameter), has demonstrated optimal performance under laboratory conditions [25,26,27]. Filler concentrations of 2–5 wt%, uniformly dispersed by sol–gel processing or in situ polymerization, have demonstrated consistent mechanical and antimicrobial results [22,34]. The material is compatible with additive manufacturing methods (SLA, DLP) and conventional processing methods [37,43].

It is important to emphasize that these considerations are theoretical and derived from in vitro studies. The photocatalytic activity of TiO_2_ in the oral cavity remains unproven, as no in vivo evidence confirms clinically significant antimicrobial effects under real oral conditions [27,44].

## 4. An Integrated Evaluation Framework of Emerging Smart Materials in Dentistry

**Figure 2:**
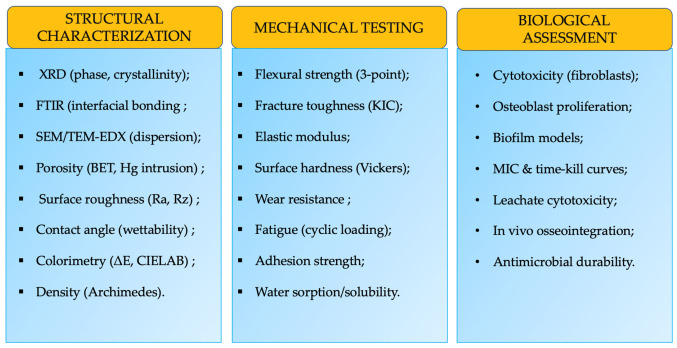
Integrated evaluation framework.

The current state-of-the-art evaluation of PMMA-based hybrid systems for removable prostheses requires a tightly integrated, multidisciplinary workflow that links structural characterization, mechanical performance, and biological function. Isolated evaluations, measuring only flexural strength or antimicrobial activity, are insufficient to predict clinical performance. This section summarizes the evaluation methodologies reported in the literature and proposes a standardized framework for future studies (Figure 2).

### 4.1. Structural Characterization: Beyond Descriptions of a Single Technique

Structural characterization should not be limited to the description of a single approach but should encompass a range of complementary analyses. X-ray diffraction (XRD) is an important method for assessing the crystalline phase and phase stability, especially for TiO_2_ and HA (crystallinity index, Ca/P ratio) [22,45].

Fourier transform infrared spectroscopy (FTIR) and Raman spectroscopy document the polymer–ceramic interfacial chemistry, confirming successful silanization (characteristic Si–O–Si and Si–O–C stretches at 1000–1200 cm^−1^), grafting or coupling activity [11,15,18,46]. Tham et al. showed that titanate coupling agents had typical Ti–O–C and P–O–C absorptions, confirming covalent incorporation [15]. High-resolution imaging, such as scanning electron microscopy (SEM) with energy-dispersive X-ray spectroscopy and transmission electron microscopy, reveals particle dispersion, interfacial wetting, and porosity architecture [12,13,18,30,31]. Agglomeration, easily observed as micron-sized clusters in a uniform dispersion of nanoparticles, has been shown to be substantially correlated with lower mechanical properties and should be assessed by image analysis.

Quantitative porosity analysis (Brunauer–Emmett–Teller, BET or mercury intrusion porosimetry) is of great importance in the design of mesoporous carriers, as pore volume and connectivity directly affect the release kinetics and water sorption behavior [16,17,20].

### 4.2. Mechanical Testing: Clinically Oriented and Comprehensive

Single-point bending strength is necessary, but not sufficient. The mechanical evaluation of removable prostheses should be thorough and include:

- Bending strength (σ, MPa): 3-point bending (typically with a 50 mm span and a crosshead speed of 5 mm/min) [5,13,32,47].
- Fracture toughness (KIC, MPa m1/2): An important property to evaluate is the resistance to crack propagation under masticatory forces [26,48].
- Modulus of elasticity (E, GPa): Stiffness and resistance to deformation [11,13].
- Surface hardness (Vickers or Knoop, HV/HK): Important for wear resistance and polishing [49,50].
- Wear resistance (volume loss, mm3 or wear rate): A metric of increasing importance for dentures and implant-supported prostheses [12,51].
- Bond strength (shear or tensile, MPa): Overdenture systems or repaired prostheses [52,53].
- Fatigue resistance: Cyclic loading (10^5^–10^6^ cycles, 50–100 N) to replicate months to years of masticatory function [7,54].
- Water sorption and solubility (%w): Critical for predicting dimensional stability and plasticity [6,7,8].
- Thermal cycling resistance: Typically, 5000–10,000 cycles of 5–55 °C with pre/post studies of mechanical and surface characteristics [22,41].

In 2025, a systematic review and network meta-analysis by Vincze et al. determined that the effects of the filling material interact with the manufacturing method (milling, heat setting, injection molding, 3D printing) and should be reported as a primary variable [5]. Regardless of filler content, milled PMMA exhibited higher flexural strength (approximately 120–140 MPa) than 3D printed (60–80 MPa) or thermally cured (70–90 MPa) composites. These results were corroborated by Fiore et al., who showed that milling gives the most homogeneous filler dispersion and the lowest porosity [47].

### 4.3. Biological Assessment: From Planktonic to Clinically Relevant Models

**Figure 3:**
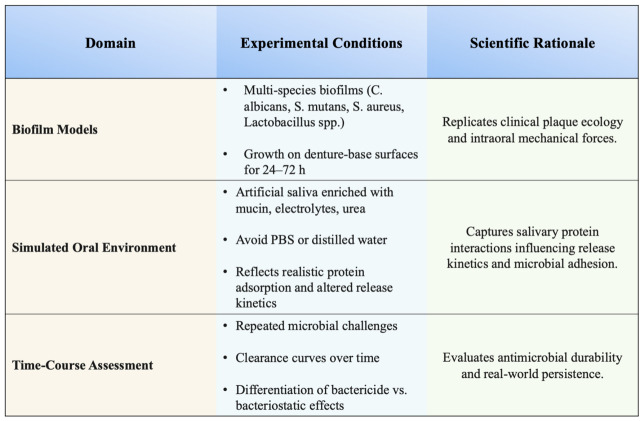
Criteria for validating antimicrobial claims in dental materials.

Biological evaluation should progress from simple planktonic assays to clinically relevant models that mimic the complexity of the oral microbiome and host response. Cytotoxicity screening using relevant oral cell lines, human gingival fibroblasts (HGF), oral keratinocytes (OKF6), and osteoblasts (MG63, Saos-2) is still a basic requirement [22,41,55,56]. International guidelines should be followed, with leachate testing to distinguish direct contact-mediated from extract-mediated toxicity (Figure 3).

Although minimum inhibitory concentration (MIC) data are valuable for a mechanistic context, biofilm reduction (log_10_ CFU/cm^2^ reduction), anti-adhesion metrics (percentage of adhesion reduction), and clearance curves over time are more representative of prosthetic and provisional media [4,9,57]. For mesoporous SiO_2_ and ion-doped HA systems, sustained release profiling (cumulative release over time, typically 1–30 days) and leachate cytotoxicity testing (evaluation of extracts on relevant cell lines) are indispensable [14,16,17]. For materials designed to interact with bone or peri-implant tissues (e.g., HA-coated temporary implant abutments), osteoblast proliferation (Alamar Blue, DNA quantification), differentiation markers (alkaline phosphatase activity, osteocalcin expression), and short-term in vivo models of osseointegration or mucocompatibility are required [2,3]. As highlighted by Natarajan et al., in vivo evidence for nanoparticle-modified acrylic resins is limited, with most studies limited to short-term (≤3 months) animal models [58,59,60].

### 4.4. The Critical Need for Standardized Aging Protocols

**Figure 4:**
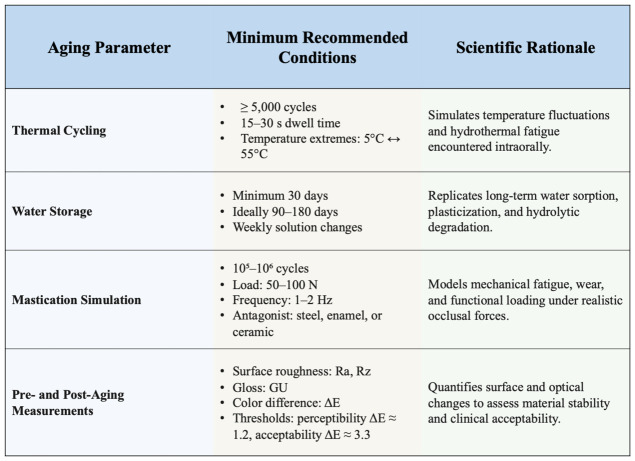
The protocols in dental materials research.

It is essential that the three domains of evaluation (structural, mechanical, biological) are linked through standardized aging and challenge protocols, so that structural changes, mechanical degradation, and biological performance are measured on the same specimens. Systematic reviews repeatedly identify heterogeneity in aging methods as a major barrier to synthesis, which is displayed in Figure 4 [21,35,61,62,63,64,65,66].

Adoption of common testing matrices and reporting templates, such as the Minimum Information for Nanomaterial Characterization (MINChar) framework adapted for dental materials, will accelerate the translation from laboratory proof of concept to clinically validated prostheses.

## 5. Discussion: Clinical Applications in Prosthodontics

The integration of HA, SiO_2_, and TiO_2_ into PMMA is particularly relevant for three categories of removable prostheses, each with distinct performance requirements.

### 5.1. Denture Bases: Surface Hardness, Wear Resistance, and Antimicrobial Performance

**Figure 5:**
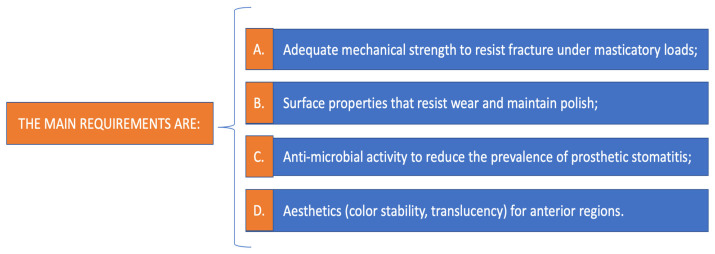
The requirements of removable dentures.

Complete and partial denture bases represent the largest clinical application for ceramic-modified PMMA (Figure 5).

The osteoconductive properties of HA are only relevant in direct contact with bone, such as at implant–prosthesis interfaces [2,3]. In conventional removable prostheses, the base of the prosthesis contacts the mucosa, not the bone. HA cannot exert osteoconductive effects through the mucosal layer. Some in vitro studies have explored HA coatings for possible mucosal compatibility, but the mechanism would relate to surface properties rather than osteoconduction, and the clinical significance remains unproven [11,12,13]. For implant-supported prostheses where modified PMMA contacts bone (e.g., healing abutments), HA-PMMA may have potential relevance based on in vitro evidence, but clinical data are lacking, and the application remains experimental [2,3]. For conventional removable prostheses that only contact the mucosa, HA-PMMA has no physiological justification.

Dual-structure RPD bases, with a nanoparticle-loaded surface layer over an unmodified PMMA core, can preserve mechanical properties while providing antibacterial activity at the surface [67,68]. Hybrid systems (e.g., HA-TiO_2_, SiO_2_, TiO_2_) demonstrate synergistic improvements in vitro [41,42].

SiO_2_-PMMA (silanized nanosilica, 1–3 wt%) has the best mechanical reinforcement to load ratio with little esthetic sacrifice and is applicable for both anterior and posterior denture bases [32,36]. The more complex and expensive mesoporous SiO_2_ systems are recommended for patients with recurrent denture stomatitis or xerostomia, where the additional complexity of the rechargeable antibacterial function is justified [16,17,67].

TiO_2_-PMMA is the most investigated system for denture bases and has consistent evidence of improved flexural and impact strength, reduced C. albicans biofilm formation, and satisfactory color stability at ≤3% weight loading [23,35,36]. The photocatalytic process is particularly suitable for maxillary dentures, as the palatal surface is exposed to ambient light.

Emerging design strategy with dual and graded structures: In 2020, Gad et al. constructed dual denture bases with a nanoparticle-loaded surface layer (Ag, TiO_2_, or ZrO_2_) over an unmodified PMMA core, maintaining overall mechanical properties while providing antibacterial activity at the surface [68]. This strategy minimizes the trade-off between biofunctionality and mechanical integrity.

Ceramic-modified PMMA for patient protection devices (RPDs) should be considered experimental until clinical trials demonstrate significant patient-centered benefits.

### 5.2. Improving Bone Contact and Reducing Complications in Implant-Supported Prostheses

In the case of implant-supported and hybrid prostheses, the prosthesis–abutment-implant interface presents additional challenges: (1) early bone contact and osseointegration of immediately loaded implants, (2) prevention of peri-implantitis by antimicrobial surfaces, and (3) reduction in friction and corrosion at the prosthesis–abutment level. HA-PMMA is the preferred modifier for implant–prosthesis interfaces, where osteoconduction is directly relevant. HA coatings on temporary implant abutments or healing capsules promote soft tissue attachment and may enhance early peri-implant bone formation [2,3]. Sasany et al. showed that Ag-doped HA-PMMA provides both osteoconductive and antimicrobial activity, addressing two crucial requirements for immediately loaded implants [69]. Silica-based coatings have been developed to enhance the bond strength between PMMA denture bases and metal or zirconium implant abutments. Akutsu-Suyama et al. reported that silica-based coatings for denture surfaces improved hardness and wear resistance while maintaining the integrity of the bond with the underlying PMMA [70].

Ti-PMMA has been studied as a coating material for implant abutments. The photocatalytic method can reduce bacterial colonization around the implant neck [71]. In 2023, Yadfout et al. systematically analyzed TiO_2_-coated dental bases and concluded that coating strategies (versus material integration) preserve the mechanical qualities of the implant abutment while providing the surface with antibacterial function [44].

### 5.3. Restorative-Prosthetic Interfaces: Managing Material Transitions

Removable temporary dentures often interact with restorative materials, such as ceramic crowns, composite restorations, metal clips, or implant abutments. These interfaces are sites of wear, corrosion, and microbial accumulation.

The interaction of metallic substructures (Co-Cr, Ti) with soft and hard tissues has been improved by coating with TiO_2_ and SiO_2_-based coatings to reduce the release of metal ions and improve tissue compatibility [37,71]. In 2019, Chen et al. demonstrated that strengthening PMMA with TiO_2_ and PEEK improved the tribological properties of the prosthesis–abutment interface, reducing the generation of wear debris [37]. Advanced design strategies, such as graded interfaces in which the filler concentration varies continuously from a ceramic-rich surface to a PMMA-rich surface, promise to minimize stress concentrations and delamination at the junctions of restorations and prostheses [72].

### 5.4. Critical Perspective

A critical appraisal of the HA-PMMA literature reveals variation in the reported results, which makes it difficult to synthesize the evidence. Aldabib and Ishak reported the best mechanical performance at ≤5 wt% HA, with reductions observed above this value. Shahkar et al. [13,30] found a synergistic reinforcement up to higher loads when combined with MWCNTs, implying that secondary reinforcement mechanisms may offset agglomeration effects. The conflicting results are partly due to methodological differences. Tham et al. showed that the effectiveness of surface modification is strongly method-dependent [15]. PMMA-grafted HA significantly improved surface hardness compared with untreated controls, while the results of titanite coupling agents were method-dependent [11]. The absence of standard dispersion protocols (sonication parameters, mixing time, polymerization methods) makes it hard to compare studies directly and impedes the development of evidence-based design guidelines. Furthermore, the clinical relevance of the osteoconductive mechanism of HA for removable prostheses is questionable, as the material contacts keratinized mucosa rather than bone in standard prosthetic applications [3,29]. This fundamental disconnect between the primary biofunctional mechanism and the context of clinical application implies that HA might be more appropriately positioned for implant–prosthesis interfaces than for prosthesis bases, a distinction often blurred in the literature [2,3].

The literature on SiO_2_-modified PMMA is contradictory, both in terms of the magnitude of the improvement in mechanical properties and the conditions under which it is achieved. Reported increases in flexural strength range from 22 to 38%. Topouzi et al. [31] attributed superior results to optimized silanization protocols, while Al-Thobity and Gad [21], in a meta-analysis, identified silanization efficiency, particle size distribution, and curing conditions as the main variables determining success. However, several studies report that even silanized particles agglomerate at loadings ≥ 5 wt%, reducing strength below the initial value [18,20], suggesting that the benefits of silanization depend on uniform dispersion, a condition rarely verified with quantitative methods in all studies. For mesoporous silica systems, the translational gap is particularly evident, while Lee et al. and Jo et al. demonstrated sustained and rechargeable antimicrobial release in vitro, Choudhury et al. subsequently showed that salivary proteins reduced silver release rates by up to 60% compared to distilled water controls, questioning the clinical relevance of release kinetics measured in simplified environments [16,17,58]. Similarly, Mukai et al. reported that multi-species biofilm models yielded significantly different results compared to planktonic assays, with many materials exhibiting antimicrobial activity in suspension but failing to prevent biofilm formation [57]. These discrepancies highlight the need for standardized test conditions that better replicate the oral environment, as current protocol heterogeneity prevents meaningful comparison and limits translation of promising laboratory results [21,57,58]. The TiO_2_-PMMA literature is highly contradictory regarding the relative efficacy of different particle morphologies and crystalline phases. Abdulrazzaq Naji et al. found that nanotubes (52% improvement in fracture toughness) performed significantly better than nanoparticles (28%), but Melo-Soares et al. showed that the effects of particle morphology were less significant when optimized dispersion procedures were used, implying that morphology-dependent effects could be masked by agglomeration [25,26,27]. The photocatalytic mechanism itself is a fundamental translational issue. For example, Giti et al. and Altarazi et al. demonstrated antimicrobial activity under controlled UV and ambient light conditions in vitro, the oral cavity being a closed, moist environment with limited light penetration, and no in vivo evidence confirms that clinically significant photocatalytic activity occurs under real oral conditions [27,35,36,44]. Furthermore, Kaurani et al. systematically analyzed the effects of surface roughness and found that poorly dispersed TiO_2_ nanoparticles increase surface roughness (Ra > 0.2 µm), which may promote, rather than prevent, biofilm retention [28]. Absence of standardized testing frameworks and the questionable clinical relevance of photocatalytic mechanisms in the oral environment collectively explain why TiO_2_-PMMA has not been adopted clinically despite extensive laboratory investigations [27,28,44].

### 5.5. Limitations and Constraints of Ceramic to Clinical Need

#### 5.5.1. HA-PMMA

Despite its well-established osteoconductive potential, the clinical translation of HA-modified PMMA for removable prostheses is constrained by three interconnected limitations [2,3,11,29]. There is a mechanical paradox, namely that low HA loadings (≤3–5 wt%) preserve flexural strength but may provide insufficient biofunctional surface density, while higher loadings (>5–7 wt%) induce stress concentration and agglomeration, reducing flexural strength by 20–35% [12,13]. Nano-HA attenuates, but does not eliminate, this effect, as its high surface energy promotes secondary agglomeration [73]. HA exhibits poor intrinsic adhesion to the hydrophobic PMMA matrix. This requires coupling agents (silanes, titanates or PMMA grafting) to prevent interfacial dehumidification, void formation and water ingress, and yet even optimized coupling does not fully reproduce the covalent bonding achievable with silanized SiO_2_ [29,73]. The main biofunctional mechanism of HA, such as osteoconduction through apatite nucleation, is of limited value in conventional prosthetic bases covering keratinized mucosa, where bone bonding is neither necessary nor desired [3]. Furthermore, pure HA possesses negligible direct antimicrobial activity, requiring ionic doping (Ag^+^, Ce^4+^, Ti^4+^), which introduces additional controlled release variables and potential cytotoxicity [14]. These constraints restrict HA to surface coatings or the application of enriched thin layers at implant–prosthesis interfaces.

#### 5.5.2. SiO_2_

Despite these advantages, mesoporous silica–PMMA systems face important limitations. Kotanidis et al. and Emam et al. have documented noticeable color changes (ΔE values exceeding the clinically acceptable threshold of 3.3) in some nanoparticle-modified PMMA formulations, especially at higher SiO_2_ loadings (≥5 wt%) or with prolonged water storage [19,64]. For removable anterior dentures, where esthetics is paramount, pore design and low loading fractions are essential.

Water sorption and solubility also increase with mesoporous SiO_2_ content. Giti et al. reported that water sorption increased from 1.8 wt% (pure PMMA) to 2.6 wt% at a 5% SiO_2_ loading, and solubility increased proportionally [8]. This plasticizing effect can reduce modulus and dimensional stability over time, especially in high-moisture oral environments [7].

Finally, antimicrobial claims for silica-based systems need to be validated with clinically realistic models. Choudhury et al. have shown that salivary media, containing proteins, electrolytes, and enzymes, significantly alter the release kinetics of silver from nanomodified PMMA, reducing release rates by up to 60% compared to distilled water controls [58]. Similarly, Mukai et al. have shown that biofilm models (multi-species, dynamic flow) produce substantially different results from planktonic assays, with many materials exhibiting antimicrobial activity in suspension but failing to prevent biofilm formation [57].

#### 5.5.3. TiO_2_

The main limitation of TiO_2_ photocatalysis is its dependence on light exposure. On open, light-exposed surfaces, such as denture bases, 3D-printed provisional dentures, maxillary dentures with palatal exposure, and ambient or dental light for polymerization can activate the photocatalytic mechanism [27,44]. However, shaded intraoral niches (subgingival implant abutments, tissue-bearing surfaces of mandibular dentures, deeply cut areas) will not benefit from photocatalysis, and alternative antimicrobial strategies (e.g., ion-doped HA or mesoporous silica) should be considered.

A second limitation concerns surface roughness. TiO_2_ nanoparticles, when not well dispersed, can aggregate, forming protrusions on the surface that increase the roughness (Ra > 0.2 µm) and actually favor biofilm retention [28]. In 2024, Fathima et al. showed that the method of TiO_2_ incorporation is essential for surface homogeneity, namely, in situ polymerization resulted in a more uniform dispersion and lower surface roughness than simple mechanical mixing [50]. Therefore, validated polishing and finishing protocols are essential for TiO_2_-modified dentures. 

### 5.6. Alternative Biomaterials

Various alternative materials have been studied to assess their use as PMMA reinforcing agents. While PEEK exhibits better fracture toughness, it has no inherent bioactivity and is still costly [74,75]. Presently, PEEK can be produced by several techniques, such as CAD/CAM milling and additive manufacturing techniques. ZrO_2_ nanoparticles (1–5 wt%) increase flexural strength but lack biofunctionality and make the material opaque [76,77,78,79,80,81]. Fiber-reinforced composites afford directional reinforcement but do not solve the biological drawbacks [2,7]. Silver nanoparticles are highly antibacterial, but they are cytotoxic and lack sufficient color stability [62,82,83,84,85,86,87,88]. ZnO nanoparticles have better colour stability and mild antimicrobial properties [89,90,91,92,93,94,95,96]. Gold nanoparticles show low antibacterial activity but improved mechanical properties [97,98]. Hybrid systems (graphene–silver, silver-doped nanotubes) exhibit synergistic advantages but require systematic optimization [56,86,99,100]. The ceramic-modified PMMA (HA, SiO_2_, TiO_2_) is of particular interest [41,42], because it provides some biofunctionalities (osteoconduction, long-term antimicrobial delivery, photocatalysis) combined with the esthetics and processability of pure PMMA. Ceramic-modified PMMA is the choice for applications requiring tissue integration or long-term antimicrobial activity, while PEEK or ZrO_2_-PMMA are the choice when fracture resistance is the main concern and biofunction is not needed [3,41,42].

## 6. Future Directions

Future research should focus on four major areas. First, systematic data on the dispersibility, printability, and post-polymerization behavior of ceramic-modified PMMA in 3D printing applications are still lacking. Studies are needed to optimize the dispersion of nanoparticles while maintaining printing resolution and interlayer adhesion. Second, practical manufacturing protocols for periodic antimicrobial reloading of refillable mesoporous SiO_2_ systems need to be developed, which could incorporate pH-sensitive or enzyme-triggered release mechanisms to reduce patient-mediated reloading. Third, preliminary synergistic effects of binary and ternary hybrid systems (HA-TiO_2_, SiO_2_-TiO_2_, HA-MWCNT) are demonstrated. Systematic optimization of formulations, ratios and integration procedures could lead to materials with improved combined mechanical, antibacterial and osteoconductive properties compared to single-phase systems. Fourth, stimuli-responsive components (piezoelectric phases, conductive nanowires) could offer functionalities beyond passive antimicrobial action, towards active detection of pH changes, microbial metabolites or occlusal loads for early diagnosis of stomatitis, denture maladjustment or parafunction.

## 7. Limitations

This review is comprehensive but acknowledges some limitations of the current evidence base. Most of the included studies are in vitro and short-term evaluations that do not reflect the multi-year clinical lifespan of removable dentures. Methodological heterogeneity of the studies, including differences in particle size, concentration, manufacturing routes, aging protocols, and outcome measurements, means that most ceramic-modifier combinations cannot be meaningfully meta-analyzed. The absence of long-term in vivo studies leaves unanswered basic questions related to nanoparticle retention, systemic release, and durability of biofunctional properties under real-world conditions. In addition, the clinical relevance of commonly used laboratory evaluation criteria (e.g., planktonic antimicrobial assays, single-point flexure tests) is questionable, as they do not adequately simulate the complex oral environment characterized by multispecific biofilms, cyclic loading, and salivary flow. Economic and practical considerations further reduce the chance of translation. The cost of synthesis, purification, surface functionalization, and quality control of nanoparticles can be considerable. Cost-effectiveness has not been systematically evaluated. It is unclear whether the additional clinical benefits are worth the additional costs in routine practice or whether modifications should be limited to high-risk patient populations. Specialized equipment, trained personnel, and validated manufacturing protocols, which may not be available in all settings, also pose a barrier to this economic viability. This review only discusses HA, silica, and titanium dioxide and does not mention other possible ceramic modifiers, such as zirconia (ZrO_2_), zinc oxide (ZnO), and gold nanoparticles. However, the evidence base of the selected ceramics is larger and more mature, supporting their focus as the main biofunctional modifiers for PMMA.

### Why Have Ceramic-Modified PMMA Systems Not Reached Clinical Practice?

Despite decades of research and hundreds of in vitro studies reporting mechanical and antimicrobial improvements, no ceramic-modified PMMA has been adopted into standard clinical practice. This failure to translate results stems from a confluence of scientific, methodological, and economic obstacles. First, there is no standardized testing protocol; direct comparison between studies is not possible, as particle sizes range from 10 nm to 10 µm, concentrations from 0.1 to 10 wt%, and manufacturing routes range from hand mixing to 3D printing [5,21,27,61]. The absence of agreement on minimum performance standards leaves regulatory approval pathways undefined. Second, the lack of long-term in vivo studies, with most studies limited to short-term animal models (≤3 months) and none longer than 12 months, raises important questions about nanoparticle retention, systemic release, and carcinogenic potential [60]. Third, the clinical relevance of commonly used laboratory evaluation criteria has not been demonstrated. Planktonic antimicrobial assays and single-point flexure tests do not mimic the complex oral environment of multispecific biofilms, cyclic loading, thermal fluctuations, and salivary flow [57,58]. Fourth, the substantial economic costs of nanoparticle synthesis, functionalization, and quality control, estimated to increase material costs by 200–500% compared with unmodified PMMA, have not been cost-effectively evaluated, and it is unclear whether the marginal clinical benefits justify the additional expense in routine practice [3,23]. Fifth, the therapeutic mechanisms of these materials are often not physiologically justified for their proposed applications: for example, the osteoconductive properties of HA are not relevant for mucosal contact denture bases, and the photocatalytic activity of TiO_2_ is questionable in the light-limited oral environment [3,27]. Taken together, these barriers indicate that the path to clinical translation does not involve further laboratory optimization of isolated properties, but rather a fundamental shift towards standardized evaluation frameworks, clinically relevant test designs, long-term safety studies, and cost-effectiveness analyses that would meet regulatory and clinical adoption requirements [5,27,60,61].

## 8. Conclusions

Polymethylmethacrylate continues to be the clinical material of choice for removable prostheses. However, its biological inertia, mechanical susceptibility and microbial permissiveness are ever more apparent limitations of modern prosthetic practice. This review synthesized the evidence for three ceramic modifiers, HA, SiO_2_ and TiO_2_, which each confer distinct biofunctional mechanisms to address these deficiencies.

HA offers osteoconductivity and tissue affinity owing to biomimetic surface chemistry and can be used in implant–prosthesis interfaces, mechanical reinforcement and refillable antimicrobial delivery systems for mesoporous formulations. TiO_2_ is a universal additive for light-exposed surfaces, providing both mechanical reinforcement and photocatalytic antimicrobial activity. The main conclusion of this review is that the future of PMMA in removable prostheses is not to be stronger, but to impart biofunctionality through surface and interface engineering. Ceramic modifiers are not passive fillers, but functional bioactive phases that actively manage the prosthesis–tissue interface.

In this review, we conceptualize PMMA as a multimodal biofunctional engineering platform rather than a basic structural polymer. The transition from the laboratory to the clinic must be accompanied by a simultaneous transition from isolated flexural strength tests and planktonic antimicrobial tests to an integrated tripartite framework that connects structural characterization (XRD, FTIR, SEM/EDX, porosity analysis), clinically oriented mechanical testing (fatigue, wear, thermal cycling, water sorption), and biological evaluation (multi-species biofilm models, simulated saliva, sustained release profiling, cytotoxicity in oral cell lines). The challenge today is not discovery, but translation, from the experimental stage to the oral cavity and from proof of concept to proven clinical benefits. Ceramic-modified PMMA should be considered experimental and should only be used in well-designed clinical trials.

## Figures and Tables

**Table 1:** Physicochemical properties that translate into complementary biofunctional mechanisms.

Property	Hydroxyapatite (HA)	Silica (SiO_2_)	Titania (TiO_2_)
Chemical formula	Ca_10_(PO_4_)_6_(OH)_2_	SiO_2_	TiO_2_
Primary biofunction	Osteoconductivity, tissue affinity	Surface reactivity, drug delivery	Photocatalytic antimicrobial
Physical attributes	Biomimetic crystallography, Ca/P ratio ~1.67	High surface area, tunable porosity (2–50 nm)	Anatase/rutile phases, bandgap ~3.2 eV
Surface chemistry	Reactive OH groups, Ca^2+^ sites	Silanol (Si–OH) groups	Ti–OH, oxygen vacancies
Interaction with PMMA	Requires coupling agents or grafting	Silanization enables covalent bonding	Silane/titanate coupling, sol–gel integration
Mechanical contribution at optimal loading	Surface hardness, elastic modulus	Flexural strength, porosity reduction	Flexural strength, impact resistance, fracture toughness
Antimicrobial mechanism	Carrier for doped ions (Ag^+^, Ce^4+^, Ti^4+^)	Mesoporous nanocarrier for sustained release	(•OH), (O_2_• −) under UV/vis light
Critical limitation	Stress concentration if agglomerated	Water sorption, color instability	Light-dependent activity
Representative references	[11,12,13,14,15]	[16,17,18,19,20,21]	[22,23,24,25,26,27,28]

**Table 2:** Effect of silanization on silica–PMMA interfacial chemistry and mechanical performance.

Feature	Unmodified SiO_2_	Silanized Silica
Surface chemistry	Hydrophilic Si–OH groups	Covalently attached organosilane with reactive C=C bonds
Compatibility with PMMA	Poor (hydrophilic vs. hydrophobic)	Excellent (covalent integration)
Dispersion state	Agglomeration, micron-scale clusters	Uniform nanoscale dispersion
Interfacial bonding	Weak van der Waals forces, mechanical interlocking	Covalent bonds between silane and methacrylate matrix
Porosity	High (interfacial voids, dewetting)	Low (dense, continuous interface)
Flexural strength	Baseline or reduced (stress concentration at agglomerates)	Increased by ~22–38%
Surface hardness	Unchanged or reduced	Increased by up to 27%
Water sorption	Increased (void-mediated uptake)	Reduced or unchanged
Color stability	Unaffected	Maintained at ≤3 wt% loading

## Data Availability

No new data were created or analyzed in this study.

## Abbreviations

The following abbreviations are used in this manuscript:

PMMA	Polymethyl methacrylate
HA	Hydroxyapatite
SiO_2_	Silica
TiO_2_	Titanium Dioxide
•OH	Hydroxyl radicals
OH^−^	Hydroxyl ion
O_2_•	Superoxide anion radical
MWCNT	Multi-Walled Carbon Nanotubes

## References

[1] Kaur H., Thakur A. (2022). Applications of Poly(Methyl Methacrylate) Polymer in Dentistry: A Review. Mater. Today Proc..

[2] Gad M.M., Fouda S.M., Al-Harbi F.A., Näpänkangas R., Raustia A. (2017). PMMA Denture Base Material Enhancement: A Review of Fiber, Filler, and Nanofiller Addition. Int. J. Nanomed..

[3] Zafar M.S. (2020). Prosthodontic Applications of Polymethyl Methacrylate (PMMA): An Update. Polymers.

[4] An S., Evans J.L., Hamlet S., Love R.M. (2023). Overview of Incorporation of Inorganic Antimicrobial Materials in Denture Base Resin: A Scoping Review. J. Prosthet. Dent..

[5] Vincze Z.É., Nagy L., Kelemen K., Cavalcante B.G.N., Gede N., Hegyi P., Bányai D., Köles L., Márton K. (2025). Milling Has Superior Mechanical Properties to Other Fabrication Methods for PMMA Denture Bases: A Systematic Review and Network Meta-Analysis. Dent. Mater..

[6] Tekale R.G., Mowade T.K., Radke U.M. (2019). Comparative Evaluation of Water Sorption of Heat-Polymerized Polymethyl Methacrylate Denture Base Resin Reinforced with Different Concentrations of Silanized Titanium Dioxide Nanoparticles: An In Vitro Study. Contemp. Clin. Dent..

[7] Alhotan A., Yates J., Zidan S., Haider J., Jurado C.A., Silikas N. (2021). Behaviour of PMMA Resin Composites Incorporated with Nanoparticles or Fibre Following Prolonged Water Storage. Nanomaterials.

[8] Giti R., Firouzmandi M., Zare Khafri N., Ansarifard E. (2022). Influence of Different Concentrations of Titanium Dioxide and Copper Oxide Nanoparticles on Water Sorption and Solubility of Heat-Cured PMMA Denture Base Resin. Clin. Exp. Dent. Res..

[9] Majrashi N.M., Al Qattan M.S., AlMubarak N.S., Alzahir K.Z., Gad M.M. (2024). Microbial Adhesion to Poly Methyl Methacrylate (PMMA) Denture Base Resins Containing Zinc Oxide (ZnO) Nanostructures: A Systematic Review of In Vitro Studies. Prosthesis.

[10] Ahmad N., Jafri Z., Khan Z.H. (2020). Evaluation of Nanomaterials to Prevent Oral Candidiasis in PMMA Based Denture Wearing Patients. A Systematic Analysis. J. Oral Biol. Craniofacial Res..

[11] Pan Y., Liu F., Xu D., Jiang X., Yu H., Zhu M. (2013). Novel Acrylic Resin Denture Base with Enhanced Mechanical Properties by the Incorporation of PMMA-Modified Hydroxyapatite. Prog. Nat. Sci. Mater. Int..

[12] Fouly A., Ibrahim A.M.M., Sherif E.-S.M., FathEl-Bab A.M.R., Badran A.H. (2021). Effect of Low Hydroxyapatite Loading Fraction on the Mechanical and Tribological Characteristics of Poly(Methyl Methacrylate) Nanocomposites for Dentures. Polymers.

[13] Aldabib J.M., Ishak Z.A.M. (2020). Effect of Hydroxyapatite Filler Concentration on Mechanical Properties of Poly (Methyl Methacrylate) Denture Base. SN Appl. Sci..

[14] Saskianti T., Wardhani K.K., Fadhila N., Wahluyo S., Dewi A.M., Nugraha A.P., Ernawati D.S., Kanawa M. (2024). Polymethylmethacrylate-Hydroxyapatite Antibacterial and Antifungal Activity against Oral Bacteria: An in Vitro Study. J. Taibah Univ. Med. Sci..

[15] Tham W.L., Chow W.S., Mohd Ishak Z.A. (2011). Effects of Titanate Coupling Agent on the Mechanical, Thermal, and Morphological Properties of Poly(Methyl Methacrylate)/Hydroxyapatite Denture Base Composites. J. Compos. Mater..

[16] Lee J.-H., El-Fiqi A., Jo J.-K., Kim D.-A., Kim S.-C., Jun S.-K., Kim H.-W., Lee H.-H. (2016). Development of Long-Term Antimicrobial Poly(Methyl Methacrylate) by Incorporating Mesoporous Silica Nanocarriers. Dent. Mater..

[17] Jo J.-K., El-Fiqi A., Lee J.-H., Kim D.-A., Kim H.-W., Lee H.-H. (2017). Rechargeable Microbial Anti-Adhesive Polymethyl Methacrylate Incorporating Silver Sulfadiazine-Loaded Mesoporous Silica Nanocarriers. Dent. Mater..

[18] Jiangkongkho P., Arksornnukit M., Takahashi H. (2018). The Synthesis, Modification, and Application of Nanosilica in Polymethyl Methacrylate Denture Base. Dent. Mater. J..

[19] Kotanidis A., Kontonasaki E., Koidis P. (2019). Color Alterations of a PMMA Resin for Fixed Interim Prostheses Reinforced with Silica Nanoparticles. J. Adv. Prosthodont..

[20] Hata K., Ikeda H., Nagamatsu Y., Masaki C., Hosokawa R., Shimizu H. (2022). Dental Poly(Methyl Methacrylate)-Based Resin Containing a Nanoporous Silica Filler. J. Funct. Biomater..

[21] Al-Thobity A.M., Gad M.M. (2021). Effect of Silicon Dioxide Nanoparticles on the Flexural Strength of Heat-Polymerized Acrylic Denture Base Material: A Systematic Review and Meta-Analysis. Saudi Dent. J..

[22] Totu E.E., Nechifor A.C., Nechifor G., Aboul-Enein H.Y., Cristache C.M. (2017). Poly(Methyl Methacrylate) with TiO_2_ Nanoparticles Inclusion for Stereolitographic Complete Denture Manufacturing—The Fututre in Dental Care for Elderly Edentulous Patients?. J. Dent..

[23] Gad M.M., Abualsaud R. (2019). Behavior of PMMA Denture Base Materials Containing Titanium Dioxide Nanoparticles: A Literature Review. Int. J. Biomater..

[24] Cierech M., Szerszeń M., Wojnarowicz J., Łojkowski W., Kostrzewa-Janicka J., Mierzwińska-Nastalska E. (2020). Preparation and Characterisation of Poly(Methyl Metacrylate)-Titanium Dioxide Nanocomposites for Denture Bases. Polymers.

[25] Abdulrazzaq Naji S., Jafarzadeh Kashi T.S., Pourhajibagher M., Behroozibakhsh M., Masaeli R., Bahador A. (2018). Evaluation of Antimicrobial Properties of Conventional Poly(Methyl Methacrylate) Denture Base Resin Materials Containing Hydrothermally Synthesised Anatase TiO_2_ Nanotubes against Cariogenic Bacteria and Candida Albicans. Iran. J. Pharm. Res..

[26] Abdulrazzaq Naji S., Behroozibakhsh M., Jafarzadeh Kashi T.S., Eslami H., Masaeli R., Mahgoli H., Tahriri M., Ghavvami Lahiji M., Rakhshan V. (2018). Effects of Incorporation of 2.5 and 5 Wt% TiO_2_ Nanotubes on Fracture Toughness, Flexural Strength, and Microhardness of Denture Base Poly Methyl Methacrylate (PMMA). J. Adv. Prosthodont..

[27] Melo-Soares V., Dos Reis A.C., Valente M.L.d.C. (2025). Optimizing Titanium Dioxide-Functionalized Polymethyl Methacrylate (PMMA-TiO_2_) for Dental Applications: A Systematic Review on Physicochemical Parameters and Antimicrobial Outcomes. Jpn. Dent. Sci. Rev..

[28] Kaurani P., Hindocha A.D., Jayasinghe R.M., Pai U.Y., Batra K., Price C. (2023). Effect of Addition of Titanium Dioxide Nanoparticles on the Antimicrobial Properties, Surface Roughness and Surface Hardness of Polymethyl Methacrylate: A Systematic Review. F1000Research.

[29] Rodríguez Lugo V., Castaño V.M., Rubio-Rosas E. (2016). Biomimetic Growth of Hydroxylapatite on SiO_2_–PMMA Hybrid Coatings. Mater. Lett..

[30] Shahkar L., Malek Khachatourian A., Nemati A. (2024). Fabrication and Characterization of PMMA Denture Base Nanocomposite Reinforced with Hydroxyapatite and Multi-Walled Carbon Nanotubes. Diam. Relat. Mater..

[31] Topouzi M., Kontonasaki E., Bikiaris D., Papadopoulou L., Paraskevopoulos K.M., Koidis P. (2017). Reinforcement of a PMMA Resin for Interim Fixed Prostheses with Silica Nanoparticles. J. Mech. Behav. Biomed. Mater..

[32] Alzayyat S.T., Almutiri G.A., Aljandan J.K., Algarzai R.M., Khan S.Q., Akhtar S., Ateeq I.S., Gad M.M. (2022). Effects of SiO_2_ Incorporation on the Flexural Properties of a Denture Base Resin: An In Vitro Study. Eur. J. Dent..

[33] Alzayyat S.T., Almutiri G.A., Aljandan J.K., Algarzai R.M., Khan S.Q., Akhtar S., Matin A., Gad M.M. (2021). Antifungal Efficacy and Physical Properties of Poly(Methylmethacrylate) Denture Base Material Reinforced with SiO_2_ Nanoparticles. J. Prosthodont..

[34] Alamgir M., Mallick A., Nayak G.C., Tiwari S.K. (2019). Development of PMMA/TiO_2_ Nanocomposites as Excellent Dental Materials. J. Mech. Sci. Technol..

[35] Giti R., Zomorodian K., Firouzmandi M., Zareshahrabadi Z., Rahmannasab S. (2021). Antimicrobial Activity of Thermocycled Polymethyl Methacrylate Resin Reinforced with Titanium Dioxide and Copper Oxide Nanoparticles. Int. J. Dent..

[36] Altarazi A., Jadaan L., McBain A.J., Haider J., Kushnerev E., Yates J.M., Alhotan A., Silikas N., Devlin H. (2024). 3D-Printed Nanocomposite Denture Base Resin: The Effect of Incorporating TiO_2_ Nanoparticles on the Growth of Candida Albicans. J. Prosthodont..

[37] Chen S.-G., Yang J., Jia Y.-G., Lu B., Ren L. (2019). TiO_2_ and PEEK Reinforced 3D Printing PMMA Composite Resin for Dental Denture Base Applications. Nanomaterials.

[38] Liao W., Zheng S., Chen S., Zhao L., Huang X., Huang L., Kang S. (2020). Surface Silanization and Grafting Reaction of Nano-Silver Loaded Zirconium Phosphate and Properties Strengthen in 3D-Printable Dental Base Composites. J. Mech. Behav. Biomed. Mater..

[39] Kumar C.A., Kumar C.R., Vamshikiran K., Deepthi G., Kumar G.N., Akhilesh M. (2019). Evaluation of Impact Strength of Dental Acrylic Resins by Incorporation of TiO_2_ Nanoparticles Using Two Different Processing Techniques. J. Contemp. Dent. Pract..

[40] Pai E., Nayak A., Hallikerimath R.B., Ruttonji Z., Astagi P., Pokale S. (2023). Comparison of Titanium Dioxide Nanoparticles and Silver Nanoparticles for Flexural Strength Once Incorporated in Heat-Cure Acrylic Denture Base Resin: An in Vitro Study. J. Indian Prosthodont. Soc..

[41] Liu Y., Wang Z., Liu X., Chen H., Huang Y., Li A., Pu Y., Guo L. (2025). Study on Mechanical Properties, Optical Properties, Cytotoxicity of TiO_2_-HAP Nanoparticles-Modified PMMA and Photodynamically Assisted Antibacterial Activity Against Candida Albicans In Vitro. Int. J. Nanomed..

[42] Cascione M., De Matteis V., Pellegrino P., Albanese G., De Giorgi M.L., Paladini F., Corsalini M., Rinaldi R. (2021). Improvement of PMMA Dental Matrix Performance by Addition of Titanium Dioxide Nanoparticles and Clay Nanotubes. Nanomaterials.

[43] Majeed H.F., Hamad T.I., Bairam L.R. (2024). Enhancing 3D-Printed Denture Base Resins: A Review of Material Innovations. Sci. Prog..

[44] Yadfout A., Asri Y., Merzouk N., Regragui A. (2023). Denture Base Resin Coated with Titanium Dioxide (TiO_2_): A Systematic Review. Int. J. Nanomed..

[45] Cierech M., Wojnarowicz J., Kolenda A., Łojkowski W., Mierzwińska-Nastalska E., Zawadzki P. (2017). Characteristics of Titanium Nano-Oxide (IV) as Potent Polymethyl Metacrylate Modifier. Prosthodontics.

[46] Elshereksi N.W., Ghazali M.J., Muchtar A., Azhari C.H. (2017). Studies on the Effects of Titanate and Silane Coupling Agents on the Performance of Poly (Methyl Methacrylate)/Barium Titanate Denture Base Nanocomposites. J. Dent..

[47] Fiore A.D., Meneghello R., Brun P., Rosso S., Gattazzo A., Stellini E., Yilmaz B. (2022). Comparison of the Flexural and Surface Properties of Milled, 3D-Printed, and Heat Polymerized PMMA Resins for Denture Bases: An in Vitro Study. J. Prosthodont. Res..

[48] Mousavi A., Aliha M.R.M., Imani D.M. (2020). Effects of Biocompatible Nanofillers on Mixed-Mode I and II Fracture Toughness of PMMA Base Dentures. J. Mech. Behav. Biomed. Mater..

[49] Waghmare A., Nair C., Shukla A.K., Chaturvedi M., Bhagat T.V., Alsubaiy E.F., Suleman G., Khader M.A., Chaturvedi S. (2024). Flexural Strength and Surface Hardness of Nanocomposite Denture Base Resins. Heliyon.

[50] Fathima A.N., Kumari K.S., Krishnan M., Raza F.B., Srinivasan R. (2024). Evaluation of Surface Homogeneity of Poly Methyl Methacrylate Denture Base Resin Fabricated by Two Different Methods of Titanium Dioxide Nanoparticles Incorporation—An in Vitro Study. J. Indian Prosthodont. Soc..

[51] Altaie S.F. (2023). Tribological, Microhardness and Color Stability Properties of a Heat-Cured Acrylic Resin Denture Base after Reinforcement with Different Types of Nanofiller Particles. Dent. Med. Probl..

[52] Gad M.M., Rahoma A., Al-Thobity A.M., ArRejaie A.S. (2016). Influence of Incorporation of ZrO_2_ Nanoparticles on the Repair Strength of Polymethyl Methacrylate Denture Bases. Int. J. Nanomed..

[53] Gad M.M., Al-Thobity A.M. (2021). The Impact of Nanoparticles-Modified Repair Resin on Denture Repairs: A Systematic Review. Jpn. Dent. Sci. Rev..

[54] Vella V., Nachiappan S., Balasubramanian A., Govindarajulu R., Preethanath R.S., Feroz S.M.A., Babu S.J., Chandolu S., Nayyar A.S. (2025). Comparative Evaluation of Flexural Strength of Acrylic Denture Base Resin Modified with Different Concentrations of Silver Nanoparticles Used as Antimicrobial Agents to Treat Denture Stomatitis: An In Vitro Study. J. Int. Clin. Dent. Res. Organ..

[55] Bhat V., John N., Shetty A., Raj V., Joseph S., Kuriakose R., Hameed S. (2021). Assessment of Flexural Strength and Cytotoxicity of Heat Cure Denture Base Resin Modified with Titanium Dioxide Nanoparticles: An In Vitro Study. J. Contemp. Dent. Pract..

[56] Bacali C., Baldea I., Moldovan M., Carpa R., Olteanu D.E., Filip G.A., Nastase V., Lascu L., Badea M., Constantiniuc M. (2020). Flexural Strength, Biocompatibility, and Antimicrobial Activity of a Polymethyl Methacrylate Denture Resin Enhanced with Graphene and Silver Nanoparticles. Clin. Oral Investig..

[57] Mukai M.-K., Iegami C.-M., Cai S., Stegun R.-C., Galhardo A.-P.-M., Costa B. (2023). Antimicrobial Effect of Silver Nanoparticles on Polypropylene and Acrylic Resin Denture Bases. J. Clin. Exp. Dent..

[58] Choudhury M., Yee N.M., Kit F.Y., Amalraj F.D. (2026). Impact of Salivary Media on Sustained Silver Release and the Structural Stability of Silver Nanomodified Polymethyl Methacrylate Denture Base Resin. J. Prosthet. Dent..

[59] Vaiyshnavi W., Jei J.B., Kumar B.M. (2022). Effect of Silver Nanoparticles on Wettability, Anti-Fungal Effect, Flexural Strength, and Color Stability of Injection-Molded Heat-Cured Polymethylmethacrylate in Human Saliva. J. Indian Prosthodont. Soc..

[60] Natarajan P., Kumar S.M., Natarajan S., Sridharan D.K.S., Narayana Kalkura D.S. (2025). Nano-Particle Coated or Impregnated Acrylic Resins in Dental Applications: A Systematic Review of in Vivo Evidence on Mechanical Properties, Biocompatibility and Clinical Performance. J. Oral Biol. Craniofac Res..

[61] Lima M., Salgado H., Correia A., Fonseca P. (2024). The Antimicrobial Effect of the Incorporation of Inorganic Substances into Heat-Cured Denture Base Resins—A Systematic Review. Prosthesis.

[62] Bangera M.K., Kotian R., Madhyastha P. (2023). Effects of Silver Nanoparticle-Based Antimicrobial Formulations on the Properties of Denture Polymer: A Systematic Review and Meta-Analysis of in Vitro Studies. J. Prosthet. Dent..

[63] Gad M.M., Abualsaud R., Alqarawi F.K., Emam A.-N.M., Khan S.Q., Akhtar S., Mahrous A.A., Al-Harbi F.A. (2021). Translucency of Nanoparticle-Reinforced PMMA Denture Base Material: An in-Vitro Comparative Study. Dent. Mater. J..

[64] Emam A.-N.M., Azmy E.A., Reda Zaki Al-kholy M., Gad M.M., Ahmed Helal M. (2023). Polymethylmethacrylate-Based Nanocomposites for Denture Base Fabrication: Impact of Nanoparticle Type and Concentration on the Color Change In Vitro. Int. J. Dent..

[65] Chee L.K.M., Bishal A.K., Bhatia H.S., Wee A.G., Takoudis C., Sukotjo C., Yuan J.C.-C. (2022). Effect of Nano Ceramic Coating on Color Perceptibility and Acceptability of Polymethylmethacrylate: In Vitro and Clinical Study. Materials.

[66] AlGhamdi M.A., Alatiyyah F.M., Almedarham R.F., Al Dawood Z.H., Alshaikhnasser F.Y., Alboryh S.Y., Khan S.Q., Abualsaud R., Gad M.M. (2024). Impact of Nanoparticle Addition on the Surface and Color Properties of Three-Dimensional (3D) Printed Polymer-Based Provisional Restorations. Nanomaterials.

[67] Abu-Shanab A.H., Sheta M.S., Al-Madboly L.A., Elbahrawy E.M.S. (2025). Effect of Adding Pomegranate Peel Extract-Loaded Mesoporous Silica on Some Mechanical and Antimicrobial Properties of Heat-Cured Acrylic Resin. Tanta Dent. J..

[68] Gad M.M.A., Abualsaud R., Al-Thobity A.M., Almaskin D.F., AlZaher Z.A., Abushowmi T.H., Qaw M.S., Akhtar S., Al-Harbi F.A. (2020). Effect of SiO_2_ Nanoparticles Addition on the Flexural Strength of Repaired Acrylic Denture Base. Eur. J. Dent..

[69] Sasany R., Jamjoom F.Z., Yilmaz B. (2025). Mechanical and Optical Properties of Additively Manufactured Denture Base Resin in Different Colors Modified with Antimicrobial Substances: An in Vitro Study. J. Prosthet. Dent..

[70] Akutsu-Suyama K., Tokuyama-Toda R., Tsutsumi-Arai C., Terada-Ito C., Iwamiya Y., Hiroi Z., Shibayama M., Satomura K. (2025). Physical Properties of New Silica-Based Denture Surface Coating. Nanomaterials.

[71] Amirabad L.M., Tahriri M., Zarrintaj P., Ghaffari R., Tayebi L. (2022). Preparation and Characterization of TiO_2_-coated Polymerization of Methyl Methacrylate (PMMA) for Biomedical Applications: In Vitro Study. Asia-Pac. J. Chem. Eng..

[72] AlQahtani G.M., AlSuhail H.S., Alqater N.K., AlTaisan S.A., Akhtar S., Khan S.Q., Gad M.M. (2023). Polymethylmethacrylate Denture Base Layering as a New Approach for the Addition of Antifungal Agents. J. Prosthodont..

[73] Sonkol N.M.A.E.-F., Kashef N.A.K., El-Segai A.A.E.-M., El-Badry A.S.M. (2024). Effect of Denture Base Acrylic Resin Containing Silver Nanoparticles on Candida Albicans Adhesion and Its Release in Artificial Saliva. Tanta Dent. J..

[74] Vetsa S.S.S.N.T., John P., Balasubramaniam M. (2025). Comparative Evaluation of Fracture Toughness and Antifungal Properties of Polymethyl Methacrylate and Polyetheretherketone Incorporated with Titanium Dioxide Nanoparticles in Different Concentrations—An in Vitro Study. J. Indian Prosthodont. Soc..

[75] Gad M.M., Al-Thobity A.M., Shahin S.Y., Alsaqer B.T., Ali A.A. (2017). Inhibitory Effect of Zirconium Oxide Nanoparticles on Candida Albicans Adhesion to Repaired Polymethyl Methacrylate Denture Bases and Interim Removable Prostheses: A New Approach for Denture Stomatitis Prevention. Int. J. Nanomed..

[76] Leão R.D.S., Moraes S.L.D.D., Gomes J.M.D.L., Lemos C.A.A., Casado B.G.D.S., Vasconcelos B.C.D.E., Pellizzer E.P. (2020). Influence of Addition of Zirconia on PMMA: A Systematic Review. Mater. Sci. Eng. C.

[77] Gad M.M., Abualsaud R., Rahoma A., Al-Thobity A.M., Al-Abidi K.S., Akhtar S. (2018). Effect of Zirconium Oxide Nanoparticles Addition on the Optical and Tensile Properties of Polymethyl Methacrylate Denture Base Material. Int. J. Nanomed..

[78] Ergun G., Sahin Z., Ataol A.S. (2018). The Effects of Adding Various Ratios of Zirconium Oxide Nanoparticles to Poly(Methyl Methacrylate) on Physical and Mechanical Properties. J. Oral Sci..

[79] Khattar A., Alsaif M.H., Alghafli J.A., Alshaikh A.A., Alsalem A.M., Almindil I.A., Alsalman A.M., Alboori A.J., Al-Ajwad A.M., Almuhanna H.M. (2022). Influence of ZrO_2_ Nanoparticle Addition on the Optical Properties of Denture Base Materials Fabricated Using Additive Technologies. Nanomaterials.

[80] Hamid S.K., Alghamdi L.A., Alshahrani F.A., Khan S.Q., Matin A., Gad M.M. (2021). In Vitro Assessment of Artificial Aging on the Antifungal Activity of PMMA Denture Base Material Modified with ZrO_2_ Nanoparticles. Int. J. Dent..

[81] Shehab M.M., Hasan R.H., Aziz R.R. (2024). The Effect of Adding ZrO_2_ Nanoparticles on the Transverse Strength and Hardness of Microwave-Cured Acrylic and Heat-Cured Acrylic Denture Base Materials. Dent. J..

[82] Palaskar J.N., Hindocha A.D., Mishra A., Gandagule R., Korde S. (2024). Evaluating the Antifungal Effectiveness, Leaching Characteristics, Flexural Strength, and Impact Strength of Polymethyl Methacrylate Added with Small-Scale Silver Nanoparticles—An in Vitro Study. J. Indian Prosthodont. Soc..

[83] Nayaki V.T., Karthigeyan S., Ali S.A., Kalarani G., Ranganathan K., Ranganathan A. (2023). Chemical Characterization of Silanized Silver Nanoparticles Impregnated in Poly (Methyl Methacrylate) Resin: An in Vitro Study. J. Indian Prosthodont. Soc..

[84] Mushannavar L.S., Nadiger R.K. (2025). Spectral Characterization of Biosynthesized Silver Nanomodified Poly(Methyl Methacrylate) Resin for Denture Applications. J. Indian Prosthodont. Soc..

[85] Mithran A., Rakhra J., Jain S.K., Chikkanna M., Gowrish S., Pillai S.G., Babu S.J., Swarnalatha C., Nayyar A.S. (2023). Comparative Evaluation of Impact Strength of Mechanically Modified Heat Polymerized Polymethyl Methacrylate (PMMA) Resin with Addition of 0.5, 1, 2 Wt% of Silver Nanoparticles (AgNPs): An In-Vitro Study. J. Orthod. Sci..

[86] Li M., Wang S., Li R., Wang Y., Fan X., Gong W., Ma Y. (2022). The Mechanical and Antibacterial Properties of Boron Nitride/Silver Nanocomposite Enhanced Polymethyl Methacrylate Resin for Application in Oral Denture Bases. Biomimetics.

[87] Kaul S., Ahmed S., Nandini V.V., Lathief J., Boruah S. (2023). Evaluation of Physical Properties of Denture Base Resins Containing Silver Nanoparticles of Aloe Barbadensis Miller, Morinda Citrifolia, and Boesenbergia Rotunda and Its Anti-Microbial Effect: An In Vitro Study. Cureus.

[88] Sun J., Wang L., Wang J., Li Y., Zhou X., Guo X., Zhang T., Guo H. (2021). Characterization and Evaluation of a Novel Silver Nanoparticles-Loaded Polymethyl Methacrylate Denture Base: In Vitro and In Vivo Animal Study. Dent. Mater. J..

[89] Sukumaran K., Ravindran S. (2024). Comparative Evaluation of the Flexural Strength of Heat-Activated Polymethyl Methacrylate Denture Base Resin With and Without 0.2% by the Weight of Silver Nanoparticles Cured by Conventional and Autoclave Methods: An In Vitro Study. Cureus.

[90] Silva I.L.I., Ferreira M.A.V., Andrade A.N.D., Nascimento P.L.A.D., Carneiro V.S.M., Mota C.C.B.D.O. (2022). Nanopartículas de Prata Em Bases de Próteses de PMMA Para Controle de Atividade Microbiana. Arq. Odontol..

[91] Parasrampuria N., Chander N.G., Chattopadhyay J. (2025). In Vitro Evaluation of the Dispersion of Nano-Polylactic Acid and Nano-Zinc Oxide Fillers in Denture Base Resins Using Spectroscopic Modalities. J. Indian Prosthodont. Soc..

[92] Cierech M., Wojnarowicz J., Kolenda A., Krawczyk-Balska A., Prochwicz E., Woźniak B., Łojkowski W., Mierzwińska-Nastalska E. (2019). Zinc Oxide Nanoparticles Cytotoxicity and Release from Newly Formed PMMA-ZnO Nanocomposites Designed for Denture Bases. Nanomaterials.

[93] Cierech M., Osica I., Kolenda A., Wojnarowicz J., Szmigiel D., Łojkowski W., Kurzydłowski K., Ariga K., Mierzwińska-Nastalska E. (2018). Mechanical and Physicochemical Properties of Newly Formed ZnO-PMMA Nanocomposites for Denture Bases. Nanomaterials.

[94] Alsmael M.A., Ibrahim S.W., Alsharbaty M.H.M., Ali S.S., Schagerl M. (2026). Enhanced Mechanical and Surface Performance of Three-Dimensionally Printed Denture Base Resin via Zinc Oxide and Samarium Oxide Nanoparticle Reinforcement. Materials.

[95] Vikram S., Chander N. (2020). Effect of Zinc Oxide Nanoparticles on the Flexural Strength of Polymethylmethacrylate Denture Base Resin. Eur. Oral Res..

[96] Szerszeń M., Cierech M., Wojnarowicz J., Górski B., Mierzwińska-Nastalska E. (2022). Color Stability of Zinc Oxide Poly(Methyl Methacrylate) Nanocomposite—A New Biomaterial for Denture Bases. Polymers.

[97] Taha E.Y., Elmahdy M.M.B., Masry S.M.M.E., Elsayed M.E. (2023). Effect of Nanogold Particles Addition on Dimensional Stability of Complete Denture Base Material: An in—Vitro Study. BMC Oral Health.

[98] Tijana A., Valentina V., Nataša T., Miloš H.-M., Atlagić Suzana G., Milica B., Yoshiyuki H., Hironori S., Ivanič A., Rebeka R. (2021). Mechanical Properties of New Denture Base Material Modified with Gold Nanoparticles. J. Prosthodont. Res..

[99] Li C., Luan J., Li S., Yu T., Han S., Chen L., Xie W., He J., Sun T. (2026). Research on the Mechanical, Antibacterial Properties and Antibacterial Mechanism of ZIF-8 and h-BN with PMMA Denture Base Resin. Dent. Mater..

[100] Hamdy T.M. (2024). Evaluation of Flexural Strength, Impact Strength, and Surface Microhardness of Self-Cured Acrylic Resin Reinforced with Silver-Doped Carbon Nanotubes. BMC Oral Health.

